# General and disease-specific pain trajectories as predictors of social and political outcomes in arthritis and cancer

**DOI:** 10.1186/s12916-018-1031-9

**Published:** 2018-04-09

**Authors:** Richard J. E. James, David A. Walsh, Eamonn Ferguson

**Affiliations:** 1 School of Psychology, University of Nottingham, University Park, Nottingham, NG7 2RD UK; 2 Arthritis UK Pain Centre and NIHR Nottingham Biomedical Research Centre, Academic Rheumatology, City Hospital, Nottingham, NG5 1PB UK

**Keywords:** Pain, Arthritis, Cancer, Social engagement, Voting, Longitudinal

## Abstract

**Background:**

While the heterogeniety of pain progression has been studied in chronic diseases, the extent to which patterns of pain progression among people in general as well as across different diseases affect social, civic and political engagement is unclear. We explore these issues for the first time.

**Methods:**

Using data from the English Longitudinal Study of Ageing, latent class growth models were used to estimate trajectories of self-reported pain in the entire cohort, and within subsamples reporting diagnoses of arthritis and cancer. These were compared at baseline on physical health (e.g. body mass index, smoking) and over time on social, civic and political engagement.

**Results:**

Very similar four-trajectory models fit the whole sample and arthritis subsamples, whereas a three-trajectory model fit the cancer subsample. All samples had a modal group experiencing minimal chronic pain and a group with high chronic pain that showed slight regression (more pronounced in cancer). Biometric indices were more predictive of the most painful trajectory in arthritis than cancer. In both samples the group experiencing the most pain at baseline reported impairments in social, civic and political engagement.

**Conclusions:**

The impact of pain differs between individuals and between diseases. Indicators of physical and psychological health differently predicted membership of the trajectories most affected by pain. These trajectories were associated with differences in engagement with social and civic life, which in turn were associated with poorer health and well-being.

**Electronic supplementary material:**

The online version of this article (10.1186/s12916-018-1031-9) contains supplementary material, which is available to authorized users.

## Background

The heterogeneous nature of pain progression in chronic diseases is a consistently observed phenomenon, with distinct patterns of pain progression linked to a number of key health indicators (e.g. obesity, smoking, joint damage) (see Table 1). Given that chronic pain, in general, impacts on a person’s social and personal life [1], it is surprising to find that associations between patterns of pain progression and any impact on social life and social/civic engagement remain incompletely defined. This paper reports for the first time how patterns of pain are linked to a wider range of social (e.g. holidays) and civic (e.g. voting, charitable work) engagement behaviours as well as standard biometric indices (e.g. body mass index (BMI)), first in a representative cohort of older English adults and then looking at this relationship in subsamples reporting a diagnosis of specific chronic conditions that may be associated with pain: arthritis and cancer.

**Table 1:** Analyses examining trajectories of pain in arthritis and cancer using latent class growth analyses (LCGAs) or Growth mixture models (GMMs), the trajectories identified and clinical and socio-demographic predictors

Paper	Sample	Trajectories	Predictors
Arthritis			
Collins, Katz, Dervan Losina [5]	OAI cohort (osteoarthritis): LCGA with up to 4th order polynomial	5 stable trajectories: severe pain (6%), high (17%) and low (32%) moderate pain, mild pain (35%), no pain (11%) (WOMAC)	Multivariate MLR, ref. no pain: female sex, depression, graded OA severity (moderate/severe +), obesity, non-white race (severe +), pre-college education (high mod./severe +)
Barnabe et al. [9]	CATCH cohort (early rheumatoid arthritis): LCGA with cubic polynomial	5 trajectories differing in baseline and speed of remission: high to medium (10%), low (19%) or remission (20%), medium to low (30%) or remission (21%) (DAS28)	Univariate analyses, Bonferroni corrected: age 50+, non-white, non-college education, unemployment, < $50K income, comorbidities, (non-remission trajectories +, High > Med/Low ++), symptom duration (mod. at baseline/High > Med+), DAS28, tender/swollen joints, ESR, CRP, physician, patient and pain score (High +), HAQ (high baseline and High > Med +)
Bastick et al. [6]	CHECK cohort (assumed early OA hip respondents only): LCGA with up to quadratic polynomial	4 trajectories: mild pain (42%), moderate decrease (17%), moderate progression (24%), severe pain (16%) (NRS)	Univariable and multivariable MLR, final model: pre-university education, use of pain transformation to cope, pain with internal hip rotation (progression/severe +), WOMAC physical function (all but mild +)
Nicholls, Thomas, van der Windt, Croft and Peat [10]	CAS-K (knee OA risk group), replicated in OAI (OA cohort): LCGA (polynomial info. reported in appendix)	5 in CAS-K: mild non-progressive (35%), progressive (28%), moderate (22%), improving (12%), severe non-improving (3%). 4 in OAI: mild, non-progressive (41%), moderate A (24%), B (19%) and C (11%), severe, non-improving (5%) (WOMAC pain)	Baseline CAS-K: differences reported on age, gender, BMI, IMD, employment, manual job, self-reported health, HADS, widespread pain, knee pain, WOMAC function, radiography, health care use inc. knee replacement
Holla et al. [7]	CHECK cohort (early symptomatic OA, knee OA only): LCGA (no info. on polynomials reported)	3 mostly stable trajectories differing in baseline and follow-up pain: good (47%), moderate (37%) and poor outcomes (16%) (WOMAC)	Uni/multivariate analyses, ref. good outcome: age, knee flexion range (poor –), BMI (mod. +), NRS, hip pain, comorbidity (mod./poor +), SF-36 vitality (mod./poor –), bony tenderness, osteophytosis, PCI resting (poor +)
Verkleij et al. [8]	Previously reported RCT (hip OA study): LCGA, linear model only	5 trajectories; three stable, two changing: mild (31%) or moderate pain (14%), alwaysin pain (14%) and regular (22%) or rapidly (19%) progressing (VAS)	Univariate MLR, ref. mild: low education, (mod./always +), BMI, morning hip stiffness, hip flexion (all bar mild +), KL, hip pain > 3 years (always/rapid +), generalised OA (always/regular prog. +), hip internal rotation (always +), back pain (all bar mild and reg prog.), trochanteric pain (all bar mild and rapid prog. +)
Bastick et al. [11]	CHECK cohort (early symptomatic OA, knee only): LCGA up to cubic polynomials	6 trajectories: constant mild (26%) or severe pain (10%), severe (5%) or moderate progression (24%), major (3%) or moderate regression (29%) (NRS)	Uni/multivariate MLR, ref. constant mild: BMI (mod. prog. and severe +), education (all bar ref. and severe –), comorbidity (severe cons. and prog. +), WOMAC physical (all bar sev., prog. and ref. +), knee joint space tenderness (prog. and mod. reg. +), painful knee flexion (maj. reg. –)
Norton et al. [13]	ERAS cohort (early (< 2 years) RA), baseline, 6 months and annual to 10 years follow-up: GMM, MAR assumed	4 trajectories: low (6%), moderate (28%) and high stable (20%), and moderate increasing (46%) (HAQ)	Univariate analyses: age, female, educational/economic disadvantage, unemployment, DAS/VAS/Larsen, comorbidity, mortality (track severity)
Wesseling et al. [4]	CHECK cohort (symptomatic knee OA), 5 years follow-up: LCGA, quadratic added	3 trajectories: marginal pain (31%), mild pain (42%) and moderate pain (26%) (with progression) (NRS, last week)	Univariate and multivariate LRs: BMI ≤ 25, subtertiary education, hip pain, comorbidities, PCI worrying and resting (marginal –)
Norton et al. [12]	ERAS and NOAR cohorts (early RA and early inflammatory polyarthritis): LCGM, polynomials added	Both cohorts showed 4 J-shaped trajectories differing in baseline severity, low (21%), moderate (32%, 33%), high (30%, 26%) and severe (16%, 20%) (HAQ)	Age, % female gender, lower SES, DAS28 increase alongside severity
Cancer			
Miaskowski et al. [14]	Sampled from breast care centres, post surgery: GMM, quadratic added, 0–6 months follow-up (NRS at shoulder/arm)	3 trajectories: no pain (42%), mild pain (24%), moderate pain (35%). All differ at baseline and remain stable	Age, white ethnicity (no pain +), education (mild pain longer time in educ.), income mild > mod. at > $100K), BMI, depression, trait anxiety (mod. +), QoL (tracks pain severity)
Miaskowski et al. [2]	Sampled from breast care centres, post surgery: GMM, quadratic added, 0–6 months follow-up (NRS at breast)	4 trajectories: no pain (32%), mild (43%), moderate (13%) and severe pain (12%). Moderate group shows progression, severe shows slight pain regression	Age (no +), trait anxiety, sleep disturbance, QoL (no –), non-white ethnicity, household income (severe –), BMI, comorbidities, QoL (severe +), CES-D depression (mod./sev +)

Percentages are rounded; as such, not all add up to 100%

Abbreviations: *BMI* body mass index, *CAS-K* Knee Clinical Assessment Study, *CATCH* Canadian Early Arthritis Cohort, *CES-D* Center for Epidemiologic Depression Scale, *CRP* C-reactive protein, *DAS* Disease Activity Score, *ERAS* Early Rheumatoid Arthritis Study, *ESR* erythrocyte sedimentation rate, *HADS* Hospital Anxiety and Depression Scale, *HAQ* Health Assessment Questionnaire, *IMD* invasive meningococcal disease, *KL* Kellgren-Lawrence, *MAR* missing at random, *MLR* multiple linear regression, *NOAR* Norfolk Arthritis Register, *NRS* numeric rating scale, *OAI* Osteoarthritis Initiative, *PCI* Pain Coping Inventory, *QoL* quality of life, *RA* rheumatoid arthritis, *SES* socio-economic status, *SF-36* Short Form 36 Health Survey, *VAS* visual analog scale, *WOMAC* Western Ontario and McMaster Universities Osteoarthritis Index

### Pain progression in arthritis and cancer

We chose to further explore pain progression and its links to social/civic engagement in arthritis and cancer, as both are common causes of disability and chronic pain across the world among older adults [1–3], with evidence for heterogeneous patterns of disease progress in both. Drawing on a range of different arthritis-specific cohorts [4–13] and cancer studies [2, 14], between three to six distinct pain progression trajectories have been identified (Table 1). Many of the trajectories identified in these analyses appear to be relatively stable; the groups differ from one another at baseline and show limited change over the course of the different time points. However, comparisons within a single cohort across disease have not been explored, so we do not know if differences in pain progression across diseases are cohort-specific. The comparison of arthritis and cancer against a population representative sample allows us to explore if similar pain progress patterns emerge and are linked to social/civic engagement in the same way or if there are disease-specific patterns.

The present analysis builds on previous findings by modelling the extent to which different patterns of pain are linked to wider social/civic engagement. We focus on six areas of social life where increased activity is associated with better physical health and subjective well-being [15–17]: holidays, non-political civic engagement (e.g. charitable work), political participation (e.g. voting), social engagement (e.g. evening classes), social activities (e.g. eating out), and the desire to engage in more of these activities but feeling that the respondent could not. If we want to target intervention at subgroups who are less likely to engage in social/civic behaviour, it is crucial that we start to understand the extent to which social engagement is limited by different patterns of pain progression. To this end, this manuscript reports latent class growth analyses from three separate samples from the English Longitudinal Study of Ageing (ELSA) [18] over a 13-year period: (1) the entire cohort of adults aged ≥ 50 years and their partners, (2) respondents diagnosed with arthritis shortly before the beginning of the ELSA and (3) respondents reporting a diagnosis of cancer at the beginning of the ELSA. Differences between trajectories were assessed using standard health and socio-demographic covariates (e.g. smoking, BMI) as well as indices of social/civic engagement.

## Methods

### Sample

Data for these analyses were taken from the ELSA [18], a cohort sample of adults over 50 (and their partners) drawn from respondents to the Health Survey for England. There have been seven waves of the study thus far, approximately separated by 2 years, covering 14 years from 2002 to 2015. In total, 18,489 respondents have participated in the core ELSA questionnaires. From Wave 3 onwards refreshment samples have been recruited, either due to attrition (Wave 4) or to include respondents who have recently turned 50 (Waves 3, 6 and 7). The data are collected by NatCen Social Research, who, in collaboration with the Institute for Fiscal Studies and University College London, lead the project. In this analysis we analysed both the core members and their partners, including those partners under 50. The data are publicly available from the UK Data Archive. The code for identifying the subsamples used in pooling data is available on request.

The first sample comprises all of the respondents who participated in Wave 1 of the ELSA (*n* = 12,099) and reported some pain data in any of the seven waves of the ELSA (*n* = 11,977).

In addition, we analysed two subsamples, one with a (recent) diagnosis of arthritis and without cancer, and one with respondents who had been diagnosed with cancer but not arthritis. The descriptive statistics for the demographic variables are reported in Additional file 1: Table S1.

The arthritis subsample comprised cases for people who participated in the ELSA from Wave 1 and reported their diagnosis to have been made in the 5 years (1998–2002) up to the beginning of the ELSA (*n* = 893). Four cases were removed from the analysis because they had not provided pain ratings at any of the time points at which they participated in the ELSA. All respondents participated in the ELSA at Wave 1, but some (*n* = 5) did not complete the pain questions at this wave (but did in subsequent waves). A portion of the arthritis sample reported that they did not know what kind of arthritis (osteoarthritis, rheumatoid arthritis or other) they had (Additional file 1: Table S1). Thirty-five respondents reported that they had a diagnosis of arthritis at Wave 2 or later, but had been diagnosed between 1998 and 2001 (at Wave 2 — as 2002 is likely to include diagnoses post initial interview) or 2002 (Wave 3 onwards).

The cancer subsample comprised persons who reported that they had been diagnosed with cancer at Wave 1 and had pain data for at least one time point (*n* = 445). Two respondents did not have pain data at Wave 1 but gave pain data at a later point. Distributions on the pain score at Wave 1 for the entire Wave 1 sample (*n* = 11,899), the arthritis (*n* = 884) and the cancer subsamples (*n* = 443) are reported in Additional file 1: Figures S1–S3.

### Measures

We coded several variables from the ELSA as described in the following subsections.

#### Coding of subsamples

Reported arthritis diagnosis and cancer were extracted from the core ELSA dataset at Wave 1. Types of arthritis (osteoarthritis, rheumatoid arthritis, other) were taken at Wave 1 and re-asked at Wave 3, as many respondents did not answer at Wave 1. Respondents with cancer were asked the initial site of malignant tumour growth (excluding minor skin cancers, which the question asked respondents to omit), number of years since diagnosis and whether they had undergone cancer treatment in the last 24 months.

#### Pain

The primary outcome variable was derived from two questions: whether the respondent was troubled by pain (Yes or No); and if so, how severe it was, rated as either mild (1), moderate (2) or severe (3), combined to generate a 4-point scale from 0 (no chronic pain) to 3 (severe chronic pain).

#### Health-centred demographics

Based on the variables used in previous analyses: age, sex, psychological distress (measured using the General Health Questionnaire (GHQ-12), administered dichotomously in the self-completion module), qualifications (from the Institute of Fiscal Studies derived variables), smoking history, current smoking status, 12 months frequency of alcohol consumption and BMI (which was recorded at the Wave 2 nurse visit) were used as indicators. Qualifications were subdivided into higher, secondary (including higher secondary, typically awarded at 18 e.g. A-levels, and lower secondary, typically awarded at 16, e.g. O-levels and General Certificates of Secondary Education), foreign/other or no qualifications.

#### Holidays

Respondents indicated (Yes = 1 or No = 0) at each wave whether they had taken a holiday in the UK, a holiday abroad and/or a daytrip or outing in the last 12 months. Responses from these questions were summed to generate a score from 0 to 3.

#### Civic, social and political engagement

Respondents were asked at each wave about their membership in a number of different types of groups (Yes = 1 and No = 0). Responses were divided into civic (membership in tenancy, residential or neighbourhood watch groups, charitable associations or church/religious groups), social (membership or educational/arts/music groups, social or sports groups or exercise/gym classes) or political (membership in a political party, trade union or environmental group) engagement based on the type of activity. These groupings were guided by the previous literature, which discriminates civic and political engagement on whether they are overtly political [16, 19].

#### Voting behaviour

The Wave 1 and Wave 3 questionnaires also asked respondents if they voted in the 2001 and 2005 UK General Elections.

#### Social activities

Two measures were derived from questions added in Wave 2 and all waves subsequently, which asked about social activities. Respondents were asked how frequently they, on a 6-point scale (1 = Twice a month or more, 6 = Never), went to the cinema, ate out, went to museums or art galleries or went to the theatre, opera or concerts. They were then asked for each activity if they wanted to engage in it more frequently but felt they could not (answered Yes/No). For engagement in social activities, responses to the four questions were reverse scored and averaged into a single score such that high scores equate to greater engagement in social activity. The four items relating to wanting to do each activity more often were summed.

### Missing data and modelling

Latent class growth models were estimated for each of the samples, with one to five linear trajectories modelled. Modelling stopped at five trajectories, because the models failed to replicate when increasing the number of random starts and iterations (whole sample), failed to converge (cancer) or began identifying small (< 5% of sample), potentially spurious classes (arthritis). Latent class growth analyses (LCGAs) were conducted using Mplus 7.11 [20]. An LCGA fits a growth model with random intercepts and slopes, using a maximum likelihood estimation procedure. Model selection was assessed using a combination of the Bayesian information criterion (BIC), entropy and overall trajectory size. The BIC is a measure of model fit derived from the sample size, the log-likelihood of the model and the number of free parameters in the model. Smaller BIC values indicate better fit. Entropy is an index of classification accuracy, with an entropy statistic > 0.8 recommended for latent class models. Once the most appropriate model had been identified with a random intercept and linear slope, polynomials were added to the model to determine whether they improved the model fit. These were assessed using BIC, because this is more conservative compared to other information criteria when comparing models with different numbers of parameters.

In addition to the statistical properties of competing models, we also considered the theoretical implications of the models we selected. In both subsamples, we expected that the most common group would be minimally or mildly troubled by pain, based on the considerable existing literature (for arthritis) (Table 1), and meta-analytic work showing that around a third of people with cancer in remission experience pain related to their disease [3]. In arthritis we further expected to observe pain progression and regression, as arthritis is characterised by worsening structural damage and increasing attempts to remedy this, for example by using exercise, medication, steroid injections or joint replacement surgery.

We accounted for missing data in all samples using a latent class dropout model [21]. Attrition might be due to a range of reasons such as death, change of address or a lack of interest in further participation. It has been noted before that the ELSA has substantial attrition [22]. Logistic regression analyses of participation in the subsequent wave (see Additional file 1: Table S2) indicate that pain was predictive of dropout in the whole sample, and in the cancer subsample but not the arthritis subsample. In the subsamples, 42.74% of the arthritis subsample and 35.82% of the cancer subsample had pain rating data at Wave 7. This compares against the 43.26% of all respondents recruited at Wave 1 who were still participating in the ELSA at Wave 7 (and the 41.46% of respondents with pain data at Waves 1 and 7).

The relationship between pain and attrition indicates that the data are not missing at random (NMAR) in any of the samples. We used a latent class dropout model to account for this. This model is an extension of the pattern-mixture approach [23] for NMAR data. Whereas pattern-mixture approaches assume that all cases of persons who drop out at a given wave have a similar response pattern, in the latent class dropout model dropout is included as a one-step covariate. This means that some of the more conservative assumptions of pattern-mixture models (such as that all cases with a specific dropout pattern have the same response pattern) are relaxed.

A subset (*n* = 119) of the arthritis respondents disputed their diagnosis at a later wave, as respondents who endorsed a chronic condition at a prior wave were asked whether they still had the condition in question. Many of these respondents either re-endorsed arthritis of the same kind at a later wave or reported multiple types of arthritis. In the latter case, it was not stated which arthritis diagnosis was being disputed. As such, we regressed failure to confirm diagnosis on the latent classes to account for this.

Lifestyle, socio-demographic and health covariates were analysed using one-way analyses of variance for baseline analyses across the latent classes, for continuous variables, χ^2^ analyses of association for categorical variables, and Fisher’s exact test where it appeared the χ^2^ approximation failed. Significance values were corrected used the *p.adjust* function in *R* (v3.1.2, R Foundation for Statistical Computing, Vienna, Austria), using Benjamini and Hochberg’s procedure to correct for the false discovery rate [24].

## Results

### Latent class growth modelling

A four-class model was the best fitting model for the whole sample and arthritis subsamples (Table 2) and a three-class model for the cancer subsample; these models were further improved by adding quadratic and cubic polynomial terms.

**Table 2:** Indices of model fit from latent class growth analyses of chronic pain in arthritis and cancer

*k*	AIC	BIC	Entropy	VLMR-LRT *p*
Whole sample				
1	177,684.561	177,839.979	–	–
2	133,343.915	133,476.949	0.871	< 0.001
3	130,255.155	130,454.705	0.848	< 0.001
4	126,764.571	127,030.638	0.851	< 0.001
+ Quadratic	124,206.071	124,501.701	0.859	
+ Cubic	123,577.681	**123,902.874**	0.869	
5	**125,988.308**	**126,320.892**	**0.901**	**< 0.001**
Arthritis				
1	14,198.301	14,298.893	–	–
2	11,316.565	11,407.577	0.702	< 0.001
3	11,200.776	11,339.689	0.702	0.008
4	11,105.289	**11,292.103**	0.757	**0.001**
+ Quadratic	10,942.055	**11,148.029**	0.841	
+ Cubic	10,914.137	**11,139.272**	**0.850**	
5	**11,084.221**	11,318.936	**0.778**	0.29
Cancer				
1	6298.329	6384.856	–	–
2	4662.729	4736.895	0.904	**< 0.001**
3	4474.875	4586.123	**0.998**	0.052
+ Quadratic	4420.884	4544.492	**0.998**	
+ Cubic	4390.708	4526.677	**0.998**	
4	4198.800	4347.131	**0.998**	0.37
5	**4128.545**	**4313.958**	0.947	0.37

In the whole sample and cancer subsample, the five class linear models failed to converge

*AIC* Akaike information criterion, *BIC* Bayesian information criterion, *VLMR-LRT* Vuong-Lo-Mendell-Rubin likelihood ratio test

Statistics highlighted in bold are the best fitting for a particular index

Standard deviations are reported in brackets for continuous variables (BMI, age, GHQ-12 and social engagement measures)

#### Whole sample

**Fig. 1:**
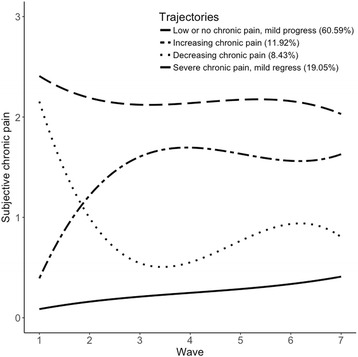
Fitted polynomial functions for each pain trajectory in the whole sample

Figure 1 displays the mean pain scores for each trajectory at each wave. Around 60% of respondents were assigned to a group of being in *low or no chronic pain, mildly progressing*. Very few respondents in this group reported being troubled by pain at Wave 1, and pain increased in later waves. However, while the growth parameters were significant, the overall size of the increase in pain was small; most respondents at each wave did not report being troubled by pain. There were two groups who reported marked pain change over the study; one reported *increasing chronic pain*, the other *decreasing chronic pain*. In both cases, the transition from being minimally troubled to substantially troubled by pain (and vice versa) occurred over the first three waves of the study before plateauing in later waves. Although the second and third order growth parameters in both cases were statistically significant, the size of the effect was relatively small. Finally, there was a trajectory of respondents reporting *severe chronic pain with mild pain regression*. These respondents reported being substantially troubled by pain at Wave 1, which reduced only a little in subsequent waves, remaining troublesome across the waves collected thus far.

#### Arthritis subsample

**Fig. 2:**
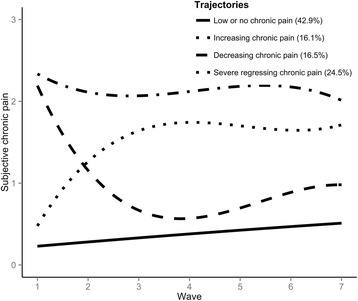
Fitted polynomial function for each of the arthritis trajectories across the seven waves

The profile of respondents in the arthritis subsample was very similar to that of the whole sample (Fig. 2). This is not surprising, as arthritis is extremely prevalent among older adults; 31.49% (*n* = 3810) of the sample at Wave 1 reported they had been diagnosed by a doctor at some point with arthritis. The modal group consisted of respondents in *low or no chronic pain*. Pain in this group remained stable over time, as none of the growth parameters were significantly different from zero (see Additional file 1: Table S3 for full growth parameter details). One trajectory of respondents displayed *increasing chronic pain*, in which respondents reported low chronic pain at baseline and increasing pain through the first three waves. A third trajectory displayed *decreasing chronic pain* with respondents reporting lower pain at follow-up than at baseline. A fourth group of respondents reported *severe receding chronic pain*; they reported severe pain that fluctuated more than the other trajectories over time, and reported less pain in later waves than at baseline (Fig. 2).

#### Cancer subsample

**Fig. 3:**
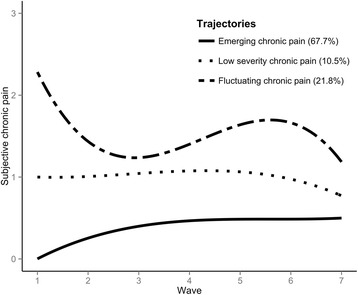
Fitted polynomial functions for each trajectory of subjective pain in the cancer sample

A three-class model was most appropriate for the cancer subsample (Table 2) (Fig. 3). Four- and five-class models displayed better BIC, but included very small classes (< 5% of sample) within the fluctuating chronic pain group that did not add explanatory value. Adding polynomials also caused the model to fail to converge, and the additional classes did not improve the entropy of the model. The trajectory to which respondents were most frequently assigned was a trajectory of *emerging chronic pain*. Unlike the arthritis analysis, this trajectory showed some mild pain progression over the waves, starting off from being unlikely to report any chronic pain, to being interposed between low and no chronic pain by the most recent wave. Unlike the arthritis analysis, where intermediate levels of pain were characterised by substantial pain change, there was a trajectory of *low severity chronic pain* in the cancer analysis. These respondents consistently reported being in chronic pain of low intensity, with no significant change in growth parameters throughout the waves. A third trajectory was characterised by *fluctuating chronic pain*. These respondents reported severe chronic pain at outset, followed by periods of increasing pain and of decreasing pain during follow-up.

### Baseline differences

Tables 3, 4 and 5 report baseline differences between trajectories for the whole sample, and the arthritis and cancer subsamples respectively.

**Table 3:** Univariate baseline differences between trajectories for the whole sample

Covariate	Low/no chronic pain (a)*N* = 7257	Increasing chronic pain (b)*N* = 1428	Decreasing chronic pain (c)*N* = 1010	Severe regressing chronic pain (d)*N* = 2282
BMI	26.36 (6.45)^b, d^	27.19 (8.16)^a^	26.67 (7.72)	27.18 (9.97)^a^
Age	63.16 (10.77)^c, d^	63.80 (10.20)^d^	64.68 (10.64)^a^	65.70 (10.37)^a, b^
GHQ-12	0.78 (1.90)^b, c, d^	1.33 (2.45)^a, d^	1.46 (2.63)^a, d^	2.22 (3.19)^a, b, c^
Sex (F)	3803 (52.40%)^a, b, c^	833 (58.33%) ^a, d^	608 (60.20%) ^a^	1455 (63.76%) ^a, b^
Non-white ethnicity	193 (2.66%)^a, b, c^	52 (3.64%)^a, c^	15 (1.49%)^a, b, d^	91 (3.99%)^a, c, d^
Alcohol:				
Non-drinker	610 (8.41%)^a, b, c^	176 (12.32%)^a, d^	132 (13.07%)^a, d^	477 (20.90%)^a, b, c^
Special occasions	1259 (17.35%)	281 (19.68%)	202 (20.00%)	573 (25.11%)
Monthly	754 (10.39%)	166 (11.62%)	119 (11.78%)	219 (9.60%)
Weekly	2381 (32.81%)	412 (28.85%)	288 (28.51%)	532 (23.31%)
Daily	1880 (25.91%)	327 (22.90%)	228 (22.57%)	380 (16.65%)
> 2 daily	328 (4.52%)	62 (4.34%)	36 (3.56%)	80 (3.51%)
Qualifications				
Higher education	1944 (26.79%)^a, b, c^	288 (20.17%)^a, d^	183 (18.12%)^a, d^	287 (12.58%)^a, b, c^
Secondary education	2111 (29.09%)	410 (28.71%)	281 (27.82%)	502 (22.00%)
Other	585 (8.06%)	126 (8.82%)	99 (9.8%)	194 (8.50%)
None	2600 (35.83%)	603 (42.23%)	446 (44.16%)	1292 (56.62%)
Ever smoked	4479 (61.72%)^b, d^	930 (65.13%)^a, d^	635 (62.87%)^d^	1576 (69.06%)^a, b, c^
Smokes now	1210 (16.67%)^d^	269 (18.84%)^d^	174 (17.23%)^d^	508 (22.26%)^a, b, c^
Holidays	1.89 (0.99)^b, c, d^	1.72 (1.00)^a, d^	1.66 (1.03)^a, d^	1.40 (1.02)^a, b, c^
Civic engagement	0.60 (0.82)^d^	0.57 (0.81)^d^	0.55 (0.81)^d^	0.46 (0.74)^a, b, c^
Social engagement	0.58 (0.73)^b, c, d^	0.51 (0.70)^a, d^	0.49 (0.69)^a, d^	0.40 (0.63)^a, b, c^
Social activities	2.09 (1.09)^b, c, d^	1.83 (1.15)^a, d^	1.72 (1.08)^a, d^	1.47 (1.13)^a, b, c^
Wishing to do more social activities	1.16 (1.38)^b, c, d^	1.45 (1.46)^a^	1.40 (1.42)^a^	1.53 (1.46)^a^
Voted, 2001	5558 (83.73%)^d^	1127 (83.36%)^d^	783 (82.77%)^d^	1555 (78.26%)^a, b, c^
Voted, 2005	3611 (88.74%)^b, c, d^	830 (85.57%)^a, d^	582 (83.5%)^a^	821 (79.94%)^a, b^
Political membership	1064 (16.57%)^b, d^	186 (14.32%)^a, d^	133 (14.70%)^d^	182 (9.67%)^a, b, c^

All comparisons are significant at the level *p* < 0.001

Standard deviations are reported in brackets for continuous variables (BMI, age, GHQ-12 and social engagement measures)

Superscripted letters denote a significant post-hoc difference between columns

**Table 4:** Univariate baseline differences between classes, arthritis LCGA

Covariate	Low/no chronic pain (a)*N* = 381	Increasing chronic pain (b)*N* = 143	Decreasing chronic pain (c)*N* = 147	Severe regressing chronic pain (d)*N* = 218	Corrected *p* (false discovery rate)
BMI	26.34 (7.55)^d^	27.48 (7.64)	26.31 (8.95)^d^	29.01 (8.44)^a, c^	0.029*
Age	64.98 (10.55)	62.90 (9.68)^c^	66.66 (10.31)^bd^	63.80 (10.60)^c^	0.028*
GHQ-12	1.09 (2.22)^d^	1.37 (2.34)^d^	1.56 (2.90)	2.29 (3.39)^a, b^	< 0.001***
Sex (F)	238 (62.47%)	77 (53.85%)	92 (62.59%)	144 (66.06%)	0.19
Non-white ethnicity	9 (2.38%)	11 (7.69%)	2 (1.36%)	5 (2.30%)	0.031*
Alcohol:					0.095
Non-drinker	49 (12.86%)	19 (13.29%)	19 (12.93%)	34 (15.60%)	
Special occasions	68 (17.85%)	26 (18.18%)	31 (21.09%)	64 (29.36%)	
Monthly	27 (7.09%)	15 (10.49%)	15 (10.20%)	22 (10.09%)	
Weekly	137 (35.96%)	47 (32.87%)	48 (32.65%)	52 (23.85%)	
Daily	83 (21.78%)	29 (20.28%)	31 (21.09%)	38 (17.43%)	
> 2 daily	17 (4.46%)	5 (3.5%)	3 (2.04%)	4 (1.83%)	
Qualifications					0.023*
Higher education	99 (25.98%)^c, d^	34 (23.78%)	20 (13.61%)^a^	36 (16.51%)^a^	
Secondary education	100 (26.25%)	41 (28.67%)	37 (25.17%)	47 (21.56%)	
Other	24 (6.3%)	11 (7.69%)	18 (12.24%)	19 (8.72%)	
None	158 (41.47%)	57 (39.86%)	72 (48.98%)	116 (53.21%)	
Ever smoked	229 (60.1%)	86 (60.14%)	96 (65.31%)	145 (66.51%)	0.39
Smokes now	48 (12.6%)	24 (16.78%)	27 (18.37%)	51 (23.39%)	0.027*
Osteoarthritis	171 (44.88%)	68 (47.55%)	66 (44.9%)	102 (46.79%)	0.93
Rheumatoid arthritis	73 (19.16%)	27 (18.88%)	25 (17.01%)	52 (23.85%)	0.43
Other arthritis	49 (12.86%)	22 (15.38%)	17 (11.56%)	21 (9.63%)	0.43
Holidays	1.90 (1.01)^c, d^	1.80 (0.99)	1.55 (1.10)^a^	1.51 (0.92)^a^	< 0.001***
Civic engagement	0.63 (0.81)	0.65 (0.82)	0.50 (0.80)	0.46 (0.76)	0.095
Social engagement	0.61 (0.72)^c^	0.58 (0.80)	0.39 (0.60)^a^	0.51 (0.73)	0.052
Social activities	2.07 (1.16)^c, d^	1.89 (1.16)	1.64 (1.15)^a^	1.62 (1.21)^a^	0.004**
Wishing to do more social activities	1.31 (1.48)	1.64 (1.51)	1.38 (1.38)	1.40 (1.49)	0.43
Voted, 2001	295 (83.33%)^d^	116 (86.57%)^d^	116 (85.93%)^d^	144 (75.39%)^a, b, c^	0.048*
Voted, 2005	196 (91.16%)^c,d^	83 (87.37%)	69 (82.14%)^a^	91 (76.47%)^a^	0.012*
Political membership	53 (15.41%)	27 (20.93%)	20 (16.26%)	25 (13.89%)	0.43

*p < 0.05, **p < 0.01, ***p < 0.001

Standard deviations are reported in brackets for continuous variables (BMI, age, GHQ-12 and social engagement measures)

Superscripted letters denote a significant post-hoc difference between columns

**Table 5:** Univariate baseline differences between classes, cancer

Covariate	Emerging chronic pain (a)*N* = 308	Low severity chronic pain (b)*N* = 48	Fluctuating chronic pain (c)*N* = 99	Corrected *p* (false discovery rate)
BMI	26.12 (6.16)	26.71 (9.02)	24.40 (10.22)	0.30
Age	66.44 (10.50)	62.52 (11.90)^c^	68.02 (11.14)^b^	0.042*
GHQ-12	0.86 (2.08)^b, c^	2.29 (3.41)^a^	1.82 (2.74)^a^	< 0.001***
Sex (F)	172 (55.84%)	35 (72.92%)	60 (60.61%)	0.18
Non-white ethnicity	1 (0.33%)	1 (2.08%)	5 (5.26%)	0.015*
Alcohol:				0.015*
Non-drinker	21 (6.82%)^c^	7 (14.58%)	15 (15.15%)^c^	
Special occasions	46 (14.94%)	7 (14.58%)	27 (27.27%)	
Monthly	38 (12.34%)	11 (22.92%)	6 (6.06%)	
Weekly	95 (30.84%)	10 (20.83%)	22 (22.22%)	
Daily	85 (27.60%)	11 (22.92%)	24 (24.24%)	
> 2 daily	72 (7.14%)	2 (4.17%)	4 (4.04%)	
Qualifications				0.006**
Higher education	103 (33.44%)^c^	10 (20.83%)	14 (14.14%)^a^	
Secondary education	74 (24.03%)	16 (33.33%)	20 (20.20%)	
Foreign/other	28 (9.09%)	6 (12.50%)	12 (12.12%)	
None	103 (33.44%)	16 (33.33%)	53 (53.54%)	
Ever smoked	198 (64.29%)	25 (52.08%)	65 (65.66%)	0.26
Current smoker	40 (12.99%)	8 (16.67%)	16 (16.16%)	0.67
Type of cancer				0.26
Lung	5 (1.62%)	1 (2.08%)	7 (7.07%)	
Breast	95 (30.84%)	18 (37.50%)	30 (30.30%)	
Colon, bowel or rectum	37 (12.01%)	6 (12.50%)	14 (14.14%)	
Lymphoma	14 (4.55%)	1 (2.08%)	2 (2.02%)	
Leukaemia	0	1 (2.08%)	1 (1.01%)	
Melanoma	27 (8.77%)	3 (6.25%)	9 (9.09%)	
Other	130 (42.21%)	18 (37.50%)	36 (36.37%)	
Years since cancer diagnosis	8.21 (8.35)	8.06 (8.20)	8.33 (8.25)	0.98
Holidays	1.87 (1.00)	1.78 (0.94)	1.70 (1.08)	0.45
Civic engagement	0.75 (0.92)	0.70 (0.81)	0.53 (0.83)	0.26
Social engagement	0.65 (0.85)	0.43 (0.62)	0.49 (0.69)	0.23
Social activities	2.21 (1.19)^c^	2.29 (1.21)^c^	1.56 (1.09)^a, b^	0.006**
Wishing to do more social activities	1.01 (1.30)	1.41 (1.50)	1.38 (1.33)	0.24
Voted, 2001	254 (89.12%)	33 (82.62%)	66 (81.48%)	0.24
Voted, 2005	158 (91.28%)^b, c^	19 (76.00%)^a^	33 (78.57%)^a^	0.031*
Political membership	36 (13.09%)	2 (4.35%)	11 (14.67%)	0.26

*p < 0.05, **p < 0.01, ***p < 0.001

Standard deviations are reported in brackets for continuous variables (BMI, age, GHQ-12 and social engagement measures)

Superscripted letters denote a significant post-hoc difference between columns

In the whole sample, respondents in the increasing and severe pain trajectories reported higher BMI than the low pain trajectory, which also tended to be younger than the trajectories with severe pain at outset. Respondents in the low/no pain trajectory were more likely to be male, less likely to abstain from alcohol and tended to have a greater number of qualifications than their peers in the other trajectories.

For health measures recorded at baseline for the arthritis subsample, high BMI and current smoking status were associated with membership of the trajectory reporting the greatest pain, as were lower alcohol consumption, having fewer qualifications and greater psychological distress. The group reporting progressing pain tended to be younger. Baseline differences in the social/civic measures in Table 4 indicated that respondents in the two trajectories reporting more pain at baseline, the decreasing chronic pain and severe regressing chronic pain groups, reported less social but not civic engagement.

In the cancer sample, respondents in the fluctuating chronic pain trajectory tended to be older than those who reported lower severity pain. Like the group reporting the most pain in arthritis, those in the cancer sample in the severe, fluctuating pain trajectory reported lower consumption of alcohol and reported less engagement in higher education. Respondents who reported low severity chronic pain reported greater levels of psychological distress relative to those who did not.

### Social life analyses

Each sample has considerable attrition over the course of the ELSA: 4966 (41.46%) of the whole sample, 163 (35.82%) of the cancer and 380 (42.74%) of the arthritis respondents had pain data at Wave 7. Non-response was associated with class in the whole sample (χ^2^(3) = 205.97, *p* < 0.001) and the arthritis subsample (χ^2^(3) = 21.57, *p* < 0.001), but not in the cancer (χ^2^ (2) = 2.44, *p* = 0.29) subsample. Uncorrected post hoc χ^2^ tests (Additional file 1: Table S4) indicated that this was due to both higher attrition in the severe pain group and lower attrition in the pain change trajectories in the whole sample, and lower attrition in the increasing pain trajectory in arthritis.

Taking this and the high level of dropout into account, differences between trajectories on measures of social life were modelled using a linear growth model, using the pattern-mixture approach. The intercept and slope of these models were then regressed on membership of the latent classes (dummy coded, with the trajectories in each sample reporting the least pain as the reference class) and age (z-scored) to account for differences between respondents of different ages in social activity.

The summary of the models shown in Table 6 shows how age and the different trajectories in pain across the three subsamples are associated with social engagement. Overall respondents engaged in fewer social activities in later waves as they aged (Additional file 1: Table S5-S8), and respondents reported increasing limitations in their ability to engage in social activities. Often the intercept and slopes of the growth models were significantly correlated, indicating that respondents reporting greater social activity at the beginning reported a faster decline over time, indicating a regression to the mean. In the whole sample, all of the trajectories reported greater limitations on each of the indicators of social and civic engagement relative to the low, mild pain progress trajectory. In addition, respondents in the severe, mild pain regression trajectory reported that their rate of engagement declined at a steeper rate for the holiday and social engagement indicators. For the indicators of holidays, civic engagement and social activities, the severe regressing (in arthritis) and fluctuating (in cancer) pain trajectory groups reported greater limitations than those in the least pain in social engagement, with lower intercepts on the growth models. Respondents in the fluctuating pain trajectory group in the cancer subsample also reported an increased desire to engage in social activities compared to the lowest pain trajectory group, but felt they could not do so. In the arthritis sample, all of the other pain trajectory groups relative to the low or no chronic pain trajectory group reported less engagement in social activities. Baseline pain was negatively associated with social engagement; respondents in the decreasing and severe regressing chronic pain trajectory groups reported taking fewer holidays and engaging in a more limited social life and social activities. The severe pain trajectory group also reported a faster decline than other groups in the frequency with which they engaged in social activities. There were no significant differences in any measures of social life between the emerging and low severity chronic pain groups in the cancer subsample.

**Table 6:** Effects of age and trajectory membership (relative to the lowest pain trajectory) for all three samples

Whole sample					
Effect	Parameter	Age	Increasing	Decreasing	Severe
Holidays	Intercept	**–**	**–**	**–**	**–**
	Slope	**–**	**–**	ns	**–**
Civic engagement	Intercept	**+**	**–**	**–**	**–**
	Slope	**–**	ns	ns	ns
Social engagement	Intercept	ns	**–**	**–**	**–**
	Slope	**–**	ns	ns	**–**
Social activities	Intercept	**–**	**–**	**–**	**–**
	Slope	**–**	ns	ns	ns
Wishing to do more social activities	Intercept	**–**	**+**	**+**	**+**
	Slope	**+**	ns	**–**	**–**
Arthritis					
Effect	Parameter	Age	Increasing	Decreasing	Severe
Holidays	Intercept	**–**	ns	**–**	**–**
	Slope	**–**	ns	ns	ns
Civic engagement	Intercept	**+**	ns	ns	**–**
	Slope	ns	ns	ns	ns
Social engagement	Intercept	ns	**–**	**–**	ns
	Slope	ns	ns	ns	**–**
Social activities	Intercept	ns	**–**	**–**	**–**
	Slope	ns	ns	ns	ns
Wishing to do more social activities	Intercept	**–**	**+**	ns	ns
	Slope	**+**	ns	ns	ns
Cancer					
Effect	Parameter	Age	Moderate	Severe	
Holidays	Intercept	**–**	ns	**–**	
	Slope	**–**	ns	ns	
Civic engagement	Intercept	ns	ns	**–**	
	Slope	ns	ns	ns	
Social engagement	Intercept	ns	ns	ns	
	Slope	ns	ns	ns	
Social activities	Intercept	**–**	ns	**–**	
	Slope	**–**	ns	ns	
Wishing to do more social activities	Intercept	ns	ns	**+**	
	Slope	ns	ns	ns	

*ns* not significant

In terms of voting behaviour, 82.6% of all ELSA participants reported voting in the 2001 General Election and 84.6% in the 2005 General Election. These figures are comparable with those provided by the British Electoral Survey (BES) for the UK population over 50, with 82.5% reporting voting in the 2001 election and 83.7% reporting voting in the 2005 election [25, 26]. At Waves 1 and 3 for the whole sample and the arthritis subsample, and Wave 3 for cancer, voting behaviour differed between the pain trajectories (see Tables 3, 4 and 5).

We used a one-sample *t* test to explore if the percentage voting in each trajectory was different from the BES 50 + average, with 82.5% as the reference for 2001 and 83.7% for 2005.

For the whole sample, respondents in the low pain trajectory with mild pain progression were slightly more likely than the BES average to vote (*t*(6637) = 2.72, *p* = 0.007), and those in the severe, mild pain regression trajectory were less likely than average to vote (*t*(1986) = −4.58, *p* < 0.001). The same pattern was observed in the 2005 election, with those in the mild pain progression trajectory more likely to vote than average (*t*(4068) = 10.18, *p* < 0.001), and those in the severe, mild pain regression trajectory less likely to vote (*t*(1026) = −3.01, *p* = 0.003).

For the arthritis sample, the results show that in the 2001 election respondents in the severe, mildly regressing trajectory were around 10% less likely to vote than the BES estimate (*t*(190) = − 2.27, *p* = 0.024). The other trajectories did not significantly differ from the estimated turnout from the BES. For the 2005 election, arthritic respondents in the severe, mildly regressing trajectory did not appear to be less likely to vote than the BES average (*t*(118) = −1.85, *p* = 0.067), whereas respondents in the low or no chronic pain trajectory were more likely to vote than the BES average (*t*(214) = 3.85, *p* < 0.001).

In the cancer sample, respondents in the emerging chronic pain trajectory were consistently more likely to vote than the BES average for persons over 50 at the 2001 (*t*(284) = 3.58, *p* < 0.001) and 2005 (*t*(171) = 3.90, *p* < 0.001) elections. No other differences were observed.

## Discussion

We identified heterogeneous groups of respondents with different trajectories of pain progression in a representative sample of older English adults, as well as subsamples of persons with arthritis and cancer. The whole sample and arthritis subsample had a common trajectory structure, but there were differences within and across the diseases studied. Thus, there is not a common experience of chronic pain. Importantly, we show for the first time that these trajectories are differentially associated with aspects of social/civic engagement, especially voting behaviour. In the whole sample and for both diseases, people in low or minimal pain were more likely to vote than the general population, and those in severe pain were less likely to. The group experiencing severe chronic pain in the whole sample and in each disease was associated with greater limitations in social engagement. In the whole sample and arthritis (but not cancer) groups, current smoking and high BMI were predictive of trajectories of persistent severe pain. Conversely, in both the arthritis and cancer groups, persistent severe pain trajectories were associated with less frequent or absence from drinking, alongside greater psychological distress.

We found that that different experiences of pain affected political engagement, which has potential implications for policymaking. Older adults are much more likely to vote than other age groups [27]. However, in the whole sample and arthritis subsamples, respondents in the severe, mildly regressing chronic pain groups were less likely to vote than the average for their age range. It is not clear why this group is less likely to vote. It may be due to levels of pain, or they may feel more politically disenfranchised. Given that many arthritis and cancer charities in the USA and the UK have political advocacy campaigns [27–29] and that political parties campaign for wider participation in the democratic process [30], it is important from a political and social policy perspective to find out why this group does not vote at the same high rate as their age group and to intervene to facilitate voting if needed. This type of civic engagement is also linked to well-being [16]; identifying routes to encourage voting and political engagement may well have health benefits. In contrast, respondents in the whole sample and arthritis subsample with low chronic pain voted at a *higher rate* than those of their age in the 2001 and 2005 UK elections, as did cancer patients with emerging pain. Again, it is unclear why these groups are more likely to vote. One speculative possibility to consider is that their illness is still mild (in terms of pain), and this may motivate them to ensure that they vote for the party they feel will provide them better pensions and with a more secure and supportive health and social care system in the future.

The trajectories were also associated with different levels of social and civic engagement, in addition to political engagement. In all three samples, social activities were limited by the presence of severe chronic pain; the group with the most severe chronic pain at baseline reported less engagement in social activities relative to those in low or minimal chronic pain, namely in the number of holidays taken, membership in civic-minded groups and frequency of social activities. In addition to this, membership of any pain group (in the whole sample and arthritis subsamples) and the severe pain group (in the cancer subsample) was associated with limitations in social and civic engagement. The severe pain groups were in some instances also associated with a more rapid decline in social engagement. Social and civic engagement (or ‘social capital’) appears to be related to a range of health and well-being outcomes [16]. At the outset, being troubled by pain appears to be associated with less social capital.

We identified commonalities between the different samples. The modal group in each sample reported minimal or low levels of chronic pain that either remained constant (arthritis) or increased only slightly (whole sample and cancer). In the whole sample and the arthritis subsample, respondents initially reporting intermediate levels of pain showed considerable change, with one trajectory characterised by pain progression and another by pain improvement. In contrast, in patients with cancer, there was a trajectory of respondents reporting low severity chronic pain with no change. Finally, all three analyses identified a trajectory of respondents who reported moderate to severe chronic pain at outset, characterised by mild regression and fluctuation. This fluctuation was more pronounced in the cancer than the whole sample and arthritis analyses. These trajectories are similar to those identified in other cohorts (Table 1), especially those studying people with osteoarthritis. However, our analyses are conducted in a general representative sample; thus, they avoid any selection bias from a disease-specific cohort.

It is especially interesting that there is a group of arthritic older adults whose chronic pain improves over time. This pain reduction goes against the commonly held belief that arthritis pain is a consequence of aging and will either get worse or remain bad. Pain improvement might be due to a relatively benign natural history for some arthritic conditions or to people adopting effective pain management strategies (medical, surgical, psychological or lifestyle) during their journey through the pain experience. Identifying the mediators of good pain prognosis might help inform people on how best to manage their arthritis pain. Interestingly however, observed improvements in subjective pain were not associated with an increase in social engagement; even after their pain improved, respondents in the decreasing chronic pain trajectory did not engage more socially. Although these respondents experienced less pain, it is unclear whether they experienced similar improvements in other measures of well-being, which might increase civic engagement, or may be improved by increased social and civic engagement. At the same time, unlike the severe pain groups, these respondents were not associated with a steeper decline in social engagement. Given the time course of the subsample used, it appears that interventions to keep people with arthritis engaged both socially and in a wider civic society should be targeted when arthritic symptoms start.

A number of limitations with this analysis ought to be considered. The pain question does not identify where respondents are troubled by pain or how widespread that pain is. Moreover, the pain question is limited in determining for how long or how often respondents have been troubled by pain. Unlike other studies in the field, this analysis does not separate osteoarthritis and rheumatoid arthritis. However, these other studies have focused on a more limited range of patient experience, focusing on biometric measures, and have also lacked a control sample against which to compare pain trajectories, especially a control sample with incident chronic pain. While our analysis focused on a general sample of arthritis patients, the results are similar to studies that have looked at different sites of osteoarthritic pain. In both this study and others that have focused on specific sites, the same biometric markers (e.g. BMI, qualifications) were predictive of the trajectory in greatest pain. The type of arthritis was unrelated to any of the trajectories, and our findings might be generalisable to arthritis pain due to a variety of pathologies. Arthritis was self-reported in the ELSA; some respondents did not identify their arthritis at Wave 1 or reported different diagnoses during consecutive waves, and more respondents reported rheumatoid arthritis than expected from its population prevalence [31]. Further studies are needed to replicate our findings with respect to social and civic activities. That being said, one of the strengths of this paper is that our analyses are conducted using a general representative sample of the English population and thus avoid any selection bias from the study of a disease-specific cohort. Thus, our findings have greater generalisability than studies of pain progression in disease-specific cohorts. Our paper also has the strength of examining pain progress over a 14-year time period so that a fuller extent of how pain changes can be observed. In addition, there is a level of imprecision associated with the year at which respondents were diagnosed; in the ELSA it is asked as either year or age of onset, both of which tended to pool around 5’s and 0’s. It is important to acknowledge that questions asking people if they have voted tend to overestimate political engagement. Surveys such as the BES are well known for overestimating voter turnout across all age groups, typically by around 10%. It is not known whether different subgroups within the ELSA might differently report voting practice.

## Conclusions

Latent class growth analyses of self-reported chronic pain in a representative sample of older English adults and subsamples with arthritis and cancer identified four, four and three trajectories respectively. A number of the health-centric variables identified in the arthritis literature discriminated between trajectories. In both disease subsamples and the whole sample, trajectories of pain progress differentially affected political, social and civic engagement. The respondents in least pain were more likely to vote than their peers in the cohort, whereas respondents in persistent pain with arthritis and across the population were less likely to vote.

## Additional file

Additional file 1: **Table S1.** Baseline health and socio-demographic characteristics of the sample. Comparisons are between the cancer and arthritis subsamples. **Table S2.** Binary logistic regression models modelling the relationship between pain and dropout or death in the Wave 1 ELSA respondents and the cancer and arthritis subsamples. **Table S3.** Model characteristics of each identified trajectory. **Table S4.** Uncorrected post hoc 2 × 2 χ^2^ tests on dropout by Wave 7 between classes. **Table S5.** Growth models for social and civic engagement variables (unstandardised). **Table S6.** Regression coefficients for age and trajectory on the intercept and slope for growth models for each index of social and civic engagement for respondents in the whole sample. **Table S7.** Regression coefficients for age and trajectory on the intercept and slope for growth models for each index of social and civic engagement for respondents with arthritis. **Table S8.** Regression coefficients for age and trajectory on the intercept and slope for growth models for each index of social and civic engagement for respondents with cancer. **Figure S1.** Histogram of the chronic pain measurement at Wave 1 for all Wave 1 ELSA respondents. **Figure S2.** Histogram of the chronic pain measurement at Wave 1 for respondents included in the arthritis subsample. **Figure S3.** Histogram of the chronic pain measurement at Wave 1 for respondents included in the cancer subsample. (DOCX 3796 kb)

## Abbreviations

BES: British Electoral Study
BIC: Bayesian Information Criterion
BMI: body mass index
ELSA: English Longitudinal Study of Ageing
GHQ-12: General Health Questionnaire
LCGA: latent class growth analysis
NMAR: not missing at random

## References

[1] Breivik H, Collett B, Ventafridda V, Cohen R, Gallacher D (2006). Survey of chronic pain in Europe: Prevalence, impact on daily life, and treatment. Eur J Pain..

[2] Miaskowski C, Cooper B, Paul SM, West C, Langford D, Levine JD, Abrams G, Hamolsky D, Dunn L, Dodd M (2012). Identification of patient subgroups and risk factors for persistent breast pain following breast cancer surgery. J Pain..

[3] van den Beuken-van Everdingen MHJ, Hochstenbach LMJ, Joosten EAJ, Tjan-Heijnen VCG, Janssen DJA (2016). Update on prevalence of pain in patients with cancer: systematic review and meta-analysis. J Pain Symptom Manage.

[4] Wesseling J, Bastick AN, ten Wolde S, Kloppenburg M, Lafeber FPJG, Bierma-Zeinstra SMA, Bijlsma JWJ (2015). Identifying trajectories of pain severity in early symptomatic knee osteoarthritis: a 5-year followup of the Cohort Hip and Cohort Knee (CHECK) study. J Rheumatol..

[5] Collins JE, Katz JN, Dervan EE, Losina E (2014). Trajectories and risk profiles of pain in persons with radiographic, symptomatic knee osteoarthritis: data from the osteoarthritis initiative. Osteoarthritis Cartilage..

[6] Bastick AN, Verkleij SPJ, Damen J, Wesseling J, Hilberdink WKHA, Bindels PJE, Bierma-Zeinstra SMA (2016). Defining hip pain trajectories in early symptomatic hip osteoarthritis — 5 year results from a nationwide prospective cohort study (CHECK). Osteoarthritis Cartilage..

[7] Holla JF, van der Leeden M, Heymans MW, Roorda LD, Bierma-Zeinstra SM, Boers M, Lems WF, Steultjens MP, Dekker J (2014). Three trajectories of activity limitations in early symptomatic knee osteoarthritis: a 5-year follow-up study. Ann Rheum Dis.

[8] Verkleij SPJ, Hoekstra T, Rozendaal RM, Waarsing JH, Koes BW, Luijsterburg PAJ, Bierma-Zeinstra SMA (2012). Defining discriminative pain trajectories in hip osteoarthritis over a 2-year time period. Ann Rheum Dis..

[9] Barnabe C, Sun Y, Boire G, Hitchon CA, Haraoui B, Thorne JC, Tin D, van der Heijde D, Curtis JR, Jamal S (2015). Heterogeneous disease trajectories explain variable radiographic, function and quality of life outcomes in the Canadian Early Arthritis Cohort (CATCH). PLoS One..

[10] Nicholls E, Thomas E, van der Windt DA, Croft PR, Peat G (2014). Pain trajectory groups in persons with, or at high risk of, knee osteoarthritis: findings from the Knee Clinical Assessment Study and the Osteoarthritis Initiative. Osteoarthritis Cartilage..

[11] Bastick AN, Wesseling J, Damen J, Verkleij SP, Emans PJ, Bindels PJ, Bierma-Zeinstra SM (2016). Defining knee pain trajectories in early symptomatic knee osteoarthritis in primary care: 5-year results from a nationwide prospective cohort study (CHECK). Br J Gen Pract..

[12] Norton S, Fu B, Scott DL, Deighton C, Symmons DP, Wailoo AJ (2014). Health Assessment Questionnaire disability progression in early rheumatoid arthritis: systematic review and analysis of two inception cohorts. Semin Arthritis Rheum..

[13] Norton S, Sacker A, Dixey J, Done J, Williams P, Young A (2013). Trajectories of functional limitation in early rheumatoid arthritis and their association with mortality. Rheumatology..

[14] Miaskowski C, Paul SM, Cooper B, West C, Levine JD, Elboim C, Hamolsky D, Abrams G, Luce J, Dhruva A (2014). Identification of patient subgroups and risk factors for persistent arm/shoulder pain following breast cancer surgery. Eur J Oncol Nurs..

[15] Chen C-C, Petrick JF (2013). Health and wellness benefits of travel experiences. J Travel Res..

[16] Helliwell JF, Putnam RD (2004). The social context of well-being. Philos Trans R Soc Lond B Biol Sci..

[17] Bath PA, Deeg D (2005). Social engagement and health outcomes among older people: introduction to a special section. Eur J Ageing..

[18] Marmot M, Oldfield Z, Clemens S, Blake M, Phelps A, Nazroo J, Steptoe A, Rogers N, Banks J, Oskala A (2016). English Longitudinal Study of Ageing: Waves 0-7, 1998-2015 [data collection]. UK Data Service.

[19] Ekman J, Amnå E (2012). Political participation and civic engagement: towards a new typology. Hum Aff..

[20] Muthén BO, Muthén LK. Mplus user’s guide. 7 Los Angeles: Muthén & Muthén; 1998-2015.

[21] Roy J (2003). Modeling longitudinal data with nonignorable dropouts using a latent dropout class model. Biometrics..

[22] Banks J, Muriel A, Smith JP (2011). Attrition and health in ageing studies: evidence from ELSA and HRS. Longit Life Course Stud.

[23] Little RJA (1993). Pattern-mixture models for multivariate incomplete data. J Am Stat Assoc..

[24] Benjamini Y, Hochberg Y (1995). Controlling the false discovery rate: a practical and powerful approach to multiple testing. J R Stat Soc Ser B Methodol..

[25] Clarke H, Sanders D, Stewart M, Whiteley PF: British Election Panel Study, 2001: Waves 1-2. In*.*: UK Data Service [SN: 4620]; 2003.

[26] Clarke H, Sanders D, Stewart M, Whiteley PF: British Election Study, 2005: Face-to-Face Survey. UK Data Service [SN: 5494], University of Essex, Colchester, UK; 2006.

[27] Arthritis Foundation. Our advocacy results. http://www.arthritis.org/advocate/our-results/. Accessed 5 June 2017.

[28] Arthritis Research UK. New MPs to fight for people with arthritis. 2015. http://www.arthritisresearchuk.org/news/press-releases/2015/may/new-mps-to-fight-for-people-with-arthritis.aspx. Accessed 5 June 2017.

[29] Cancer Research UK. Cross cancer out. http://www.cancerresearchuk.org/cross-cancer-out. Accessed 5 June 2017.

[30] House of Commons Political and Constitutional Reform Committee (2014). Voter engagement in the UK: Fourth Report of Session 2014-2015.

[31] Alamanos Y, Drosos AA (2005). Epidemiology of adult rheumatoid arthritis. Autoimmun Rev..

