# Supplementary material for: General and disease-specific pain trajectories as predictors of social and political outcomes in arthritis and cancer
Source: BMC Med. 2018 Apr 9;16:51. doi: 10.1186/s12916-018-1031-9 (PMC5890349; doi:10.1186/s12916-018-1031-9)
Supplement: Supplementary file 1 — Table S1. Baseline health and socio-demographic characteristics of the sample. Comparisons are between the cancer and arthritis subsamples. Table S2. Binary logistic regression models modelling the relationship between pain and dropout or death in the Wave 1 ELSA respondents and the cancer and arthritis subsamples. Table S3. Model characteristics of each identified trajectory. Table S4. Uncorrected post hoc 2 × 2 χ2 tests on dropout by Wave 7 between classes. Table S5. Growth models for social and civic engagement variables (unstandardised). Table S6. Regression coefficients for age and trajectory on the intercept and slope for growth models for each index of social and civic engagement for respondents in the whole sample. Table S7. Regression coefficients for age and trajectory on the intercept and slope for growth models for each index of social and civic engagement for respondents with arthritis. Table S8. Regression coefficients for age and trajectory on the intercept and slope for growth models for each index of social and civic engagement for respondents with cancer. Figure S1. Histogram of the chronic pain measurement at Wave 1 for all Wave 1 ELSA respondents. Figure S2. Histogram of the chronic pain measurement at Wave 1 for respondents included in the arthritis subsample. Figure S3. Histogram of the chronic pain measurement at Wave 1 for respondents included in the cancer subsample. (DOCX 3796 kb) [file 12916_2018_1031_MOESM1_ESM.docx]

Table S1
Baseline health and socio-demographic characteristics of the sample. Comparisons are between the cancer and arthritis subsamples.

| Variable | Whole Wave 1 sample (*n* = 11,977) | Cancer (mean (s.d.) or count (%)) (*n* = 455) | Arthritis (mean (s.d.) or count (%)) (*n* = 889) | Corrected *p* values (FDR) |
| --- | --- | --- | --- | --- |
| Age | 63.84 (10.66) | 66.37 (10.87) | 63.63 (10.44) | .027 * |
| Sex (Female) | 6,699 (55.93%) | 267 (58.68%) | 551 (61.98%) | .31 |
| GHQ-12 | 1.18 (2.39) | 1.22 (2.46) | 1.51 (2.72) | .087 |
| BMI | 26.65 (7.54) | 25.86 (7.42) | 27.11 (8.09) | .040 * |
| Have ever smoked (Y) | 7,620 (63.62%) | 288 (63.30%) | 556 (62.54%) | .85 |
| Current smoker (Y) | 2,161 (18.04%) | 64 (14.07%) | 150 (16.87%) | .27 |
| Current drinking status: |  |  |  | .008 ** |
| Non drinker | 1,395 (11.65%) | 43 (9.45%) | 121 (13.61%) |  |
| Special occasions | 2,315 (19.33%) | 80 (17.58%) | 189 (21.26%) |  |
| 1-2 times per month | 1,258 (10.50%) | 55 (12.09%) | 79 (8.89%) |  |
| 1-2 times per week | 3,613 (30.17%) | 127 (27.91%) | 284 (31.95%) |  |
| Daily or almost daily | 2,815 (23.5%) | 120 (26.37%) | 121 (13.61%) |  |
| Twice a day or more | 506 (4.22%) | 28 (6.15%) | 29 (3.26%) |  |
| Qualifications: |  |  |  | .027 * |
| Higher education | 2,702 (22.56%) | 127 (27.91%) | 189 (21.26%) |  |
| Secondary education | 3,304 (27.59%) | 110 (24.18%) | 225 (25.31%) |  |
| Other/foreign quals | 1,004 (8.38%) | 48 (10.11%) | 72 (8.1%) |  |
| No qualifications | 4,941 (41.25%) | 172 (37.80%) | 403 (45.33%) |  |
| Type of arthritis: |  |  |  | N/A |
| Osteoarthritis | - | - | 407 (45.78%) |  |
| Rheumatoid arthritis | - | - | 117 (13.16%) |  |
| Other arthritis | - | - | 109 (12.26%) |  |
| Initial site of cancer: |  |  |  | N/A |
| Lung | - | 13 (2.86%) | - |  |
| Breast | - | 143 (31.43%) | - |  |
| Colon, bowel or rectum | - | 57 (12.53%) | - |  |
| Lymphoma | - | 17 (3.74%) | - |  |
| Leukaemia | - | 2 (0.44%) | - |  |
| Melanoma | - | 39 (8.57%) | - |  |
| Other | - | 184 (40.44%) | - |  |
| Cancer treatment in 24m | - | 200 (43.96%) | - |  |
| Cancer duration (years) | - | 8.22 (8.29) | - |  |
| Holidays | 1.76 (1.02) | 1.83 (1.01) | 1.73 (1.03) | .17 |
| Civic Engagement | 0.57 (0.81) | 0.70 (0.90) | 0.57 (0.80) | .027 * |
| Social engagement | 0.53 (0.71) | 0.60 (0.80) | 0.55 (0.72) | .32 |
| Social activities | 1.92 (1.13) | 2.10 (1.20) | 1.87 (1.18) | .027 * |
| Wishing to do more social activities | 1.28 (1.41) | 1.12 (1.34) | 1.41 (1.47) | .027 * |

Note: *p* values - * = *p* < .05, ** *p* < .01

Table S2

Binary logistic regression models modelling the relationship between pain and dropout or death in the Wave 1 ELSA respondents, and the cancer and arthritis samples.

| Sample : Wave | *b* | *se* | *p* | |
| --- | --- | --- | --- | --- |
| Measure – Death by end of Wave 5 (using index file) | | | |  |
| All – Wave 1 pain | 0.203 | 0.020 | <.001 *** | |
| Arthritis – Wave 1 pain | 0.171 | 0.077 | .027 * | |
| Cancer – Wave 1 pain | 0.203 | 0.101 | .045 * | |
| Measure – Dropout in wave following pain measurement | | | |  |
| Wave 1 resps – Wave 1 pain | 0.090 | 0.020 | <.001 *** | |
| Wave 1 resps – Wave 2 pain | 0.108 | 0.026 | <.001 *** | |
| Wave 1 resps– Wave 3 pain | 0.144 | 0.028 | <.001 *** | |
| Wave 1 resps– Wave 4 pain | 0.062 | 0.035 | .079 | |
| Wave 1 resps – Wave 5 pain | 0.194 | .036 | <.001 *** | |
| Wave 1 resps – Wave 6 pain | 0.099 | 0.035 | .005 ** | |
| Arthritis – Wave 1 pain | 0.069 | 0.077 | .38 | |
| Arthritis – Wave 2 pain | 0.027 | 0.088 | .76 | |
| Arthritis – Wave 3 pain | 0.166 | 0.094 | .078 | |
| Arthritis – Wave 4 pain | -0.062 | 0.120 | .60 | |
| Arthritis – Wave 5 pain | 0.176 | 0.150 | .24 | |
| Arthritis – Wave 6 pain | 0.455 | 0.307 | .14 | |
| Cancer – Wave 1 pain | 0.306 | 0.105 | .004 ** | |
| Cancer – Wave 2 pain | 0.317 | 0.137 | .020 * | |
| Cancer – Wave 3 pain | 0.056 | 0.167 | .74 | |
| Cancer – Wave 4 pain | -0.030 | 0.190 | .88 | |
| Cancer – Wave 5 pain | -0.081 | 0.182 | .66 | |
| Cancer – Wave 6 pain | 0.131 | 0.175 | .46 | |

Table S3

Model characteristics of each identified trajectory

|  |  | Intercept | Slopes | | |
| --- | --- | --- | --- | --- | --- |
| Trajectory | *N* | Intercept (*se*) | Linear (*se*) | Quadratic (*se*) | Cubic (*se*) |
| Whole sample |  |  |  |  |  |
| Low or no chronic pain, mild progress | 7,257 | 0.086 (0.004) *** | 0.090 (0.014) *** | -0.018 (0.007) ** | 0.002 (0.001) * |
| Increasing chronic pain | 1,428 | 0.393 (0.019) *** | 1.094 (0.062) *** | -0.292 (0.029) *** | 0.024 (0.003) *** |
| Decreasing chronic pain | 1,010 | 2.154 (0.019) *** | -1.599 (0.065) *** | 0.481 (0.029) *** | -0.042 (0.003) *** |
| Severe regressing chronic pain | 7,257 | 2.408 (0.018) *** | -0.315 (0.036) *** | 0.108 (0.017) *** | -0.011 (0.002) *** |
| Arthritis |  |  |  |  |  |
| Low or no chronic pain, stable | 381 | 0.229 (0.025) *** | 0.053 (0.073) | -0.001 (0.033) | 0.000 (0.004) |
| Increasing chronic pain | 143 | 0.477 (0.055) *** | 1.034 (0.151) *** | -0.270 (0.068) *** | 0.022 (0.008) ** |
| Decreasing chronic pain | 147 | 2.192 (0.057) *** | -1.366 (0.168) *** | 0.356 (0.080) *** | -0.027 (0.009) ** |
| Severe regressing chronic pain, | 218 | 2.336 (0.043) *** | -0.342 (0.120) ** | 0.132 (0.053) * | -0.014 (0.006) * |
| Cancer |  |  |  |  |  |
| Emerging chronic pain | 308 | 0.001 (0.001) | 0.317 (0.055) *** | -0.069 (0.026) ** | 0.005 (0.003) |
| Low severity chronic pain. | 48 | 1.000 (0.002) *** | -0.020 (0.178) | 0.033 (0.086) | -0.006 (0.010) |
| Fluctuating chronic pain | 99 | 2.283 (0.045) *** | -1.269 (0.172) *** | 0.469 (0.081) *** | -0.048 (0.010) *** |

Note: n is calculated from each respondent’s most likely latent class.

Table S4

Uncorrected post-hoc 2 x 2 chi-square tests on dropout by W7 between classes.

| Whole Sample |  |  |  |  |
| --- | --- | --- | --- | --- |
|  | Low/no chronic pain (43.08%) | Increasing chronic pain (48.95%) | Decreasing chronic pain (47.72%) | Severe chronic pain (28.88%) |
| Low/no chronic pain | - |  |  |  |
| Increasing chronic pain | <.001 *** | - |  |  |
| Decreasing chronic pain | .006 ** | .578 | - |  |
| Severe chronic pain | <.001 *** | <.001 *** | <.001 *** | - |
| Arthritis |  |  |  |  |
|  | Low/no chronic pain (42.52%) | Increasing chronic pain (58.74%) | Decreasing chronic pain (40.14%) | Severe chronic pain (34.40%) |
| Low/no chronic pain | - |  |  |  |
| Increasing chronic pain | .001 ** | - |  |  |
| Decreasing chronic pain | .690 | .002 ** | - |  |
| Severe chronic pain | .062 | <.001 *** | .316 | - |

Table S5

Growth models for social and civic engagement variables (unstandardized)

| Effect | *b* | *se* | *P* |
| --- | --- | --- | --- |
| Whole sample |  |  |  |
| *Holidays (RMSEA = .022, CFI = .984, SRMR = .075)* | | | |
| Intercept | 2.032 | 0.015 | <.001 |
| Slope | -0.051 | 0.003 | <.001 |
| Intercept with slope | -0.011 | 0.002 | <.001 |
| *Civic engagement (RMSEA = .021, CFI = .987, SRMR = .023)* | | | |
| Intercept | 0.716 | 0.013 | <.001 |
| Slope | -0.007 | 0.002 | <.001 |
| Intercept with slope | -0.014 | 0.001 | <.001 |
| *Social engagement (RMSEA = .024, CFI = .974, SRMR = .036)* | | | |
| Intercept | 0.658 | 0.011 | <.001 |
| Slope | -0.007 | 0.002 | .001 |
| Intercept with slope | -0.012 | 0.001 | <.001 |
| *Social activities (RMSEA = .028, CFI = .981, SRMR = .045)* | | | |
| Intercept | 2.202 | 0.019 | <.001 |
| Slope | -0.017 | 0.004 | <.001 |
| Intercept with slope | -0.034 | 0.004 | <.001 |
| *Wishing to do more social activities (RMSEA = .020, CFI = .981, SRMR = .023)* | | | |
| Intercept | 1.125 | 0.024 | <.001 |
| Slope | -0.066 | 0.006 | <.001 |
| Intercept with slope | -0.082 | 0.005 | <.001 |
| Arthritis |  |  |  |
| *Holidays (RMSEA = .02, CFI = .987, SRMR = .076)* | | | |
| Intercept | 2.074 | 0.058 | <.001 *** |
| Slope | -0.059 | 0.012 | <.001 *** |
| Intercept with slope | -0.008 | 0.007 | .24 |
| *Civic* *engagement (RMSEA = .013, CFI = .994, SRMR = .036)* | | | |
| Intercept | 0.669 | 0.050 | <.001 *** |
| Slope | -0.005 | 0.009 | .58 |
| Intercept with slope | -0.010 | 0.004 | .012 * |
| *Social* *engagement (RMSEA = .023, CFI = .974, SRMR = .069)* | | | |
| Intercept | 0.668 | 0.044 | <.001 *** |
| Slope | -0.003 | 0.010 | .80 |
| Intercept with slope | -0.018 | 0.004 | <.001 *** |
| *Social* *activities (RMSEA = .041, CFI = .956, SRMR = .079)* | | | |
| Intercept | 2.199 | 0.080 | <.001 *** |
| Slope | 0.009 | 0.019 | .65 |
| Intercept with slope | -0.063 | 0.017 | <.001 *** |
| *Wish to do more social activities (RMSEA = .03, CFI = .950, SRMR = .073)* | | | |
| Intercept | 1.354 | 0.101 | <.001 *** |
| Slope | -0.099 | 0.026 | <.001 *** |
| Intercept with slope | -0.056 | 0.026 | .032 * |
| Cancer |  |  |  |
| *Holidays (RMSEA = .032) CFI = .965, SRMR = .133)* | | | |
| Intercept | 2.130 | 0.079 | <.001 *** |
| Slope | -0.064 | 0.015 | <.001 *** |
| Intercept with slope | -0.019 | 0.011 | .080 |
| *Civic engagement (RMSEA = .019, CFI = .990, SRMR = .099)* | | | |
| Intercept | 0.937 | 0.076 | <.001 *** |
| Slope | -0.014 | 0.013 | .28 |
| Intercept with slope | -0.028 | 0.008 | <.001 *** |
| *Social engagement (RMSEA = .011, CFI = .996, SRMR = .201)* | | | |
| Intercept | 0.758 | 0.062 | <.001 |
| Slope | -0.003 | 0.014 | .83 |
| Intercept with slope | -0.018 | 0.007 | .007 ** |
| *Social activities (RMSEA = 0.00, CFI = 1.00, SRMR = .071)* | | | |
| Intercept | 2.512 | 0.103 | <.001 *** |
| Slope | -0.072 | 0.021 | .001 ** |
| Intercept with slope | -0.063 | 0.020 | .001 ** |
| *Wish to do more social activities (RMSEA = 0.00, CFI = 1.00, SRMR = 0.237)* | | | |
| Intercept | 1.122 | 0.123 | <.001 *** |
| Slope | -0.088 | 0.030 | .009 ** |
| Intercept with slope | -0.071 | 0.035 | .041 * |

Note: *p* values - * = *p* < .05, ** = *p* < .01, *** = *p* < .001. Models are adjusted for age (converted into *z* scores to allow meaningful interpretation)

Table S6

Regression coefficients for age and trajectory on the intercept and slope for growth models for each index of social and civic engagement for respondents in the whole sample.

| Effect |  |  | | Trajectories |  |
| --- | --- | --- | --- | --- | --- |
| Arthritis | Age | Increasing chronic pain | | Decreasing chronic pain | Severe chronic pain, regressing |
| *Holidays* |  |  | |  |  |
| Intercept | -0.177 (0.009) *** | -0.198 (0.026) *** | | -0.249 (0.030) *** | -0.405 (0.023) *** |
| Slope | -0.042 (0.002) *** | -0.015 (0.005) ** | | 0.000 (0.006) | -0.016 (0.005) ** |
| *Civic* *engagement* |  |  | |  |  |
| Intercept | 0.111 (0.008) *** | -0.068 (0.022) ** | | -0.064 (0.026) * | -0.127 (0.019) *** |
| Slope | -0.005 (0.002) ** | 0.004 (0.004) | | -0.001 (0.005) | -0.004 (0.004) |
| *Social engagement* | |  | |  |  |
| Intercept | -0.006 (0.007) | -0.104 (0.019) *** | | -0.098 (0.002) *** | -0.156 (0.017) *** |
| Slope | -0.008 (0.002) *** | -0.002 (0.003) | | 0.000 (0.005) | -0.010 (0.004) * |
| *Social activities* |  |  | |  |  |
| Intercept | -0.080 (0.013) *** | -0.235 (0.033) *** | | -0.326 (0.038) *** | -0.537 (0.032) *** |
| Slope | -0.032 (0.003) *** | -0.012 (0.008) | | 0.017 (0.009) | 0.001 (0.008) |
| *Wish to do more social activities* | | |  |  |  |
| Intercept | -0.212 (0.017) *** | 0.351 (0.044) *** | | 0.308 (0.050) *** | 0.413 (0.043) *** |
| Slope | 0.018 (0.005) *** | -0.021 (0.012) | | -0.042 (0.013) *** | -0.037 (0.012) *** |

Note: * = *p <* .05, ** = *p* < .01, *** = *p* < .001

Table S7

Regression coefficients for age and trajectory on the intercept and slope for growth models for each index of social and civic engagement for respondents with arthritis.

| Effect |  | |  | | Trajectories |  |
| --- | --- | --- | --- | --- | --- | --- |
| Arthritis | Age | | Increasing chronic pain | | Decreasing chronic pain | Severe chronic pain, regressing |
| *Holidays* |  | |  | |  |  |
| Intercept | -0.175 (0.032) *** | | -0.163 (0.087) | | -0.340 (0.087) *** | -0.410 (0.078) *** |
| Slope | -0.048 (0.009) *** | | -0.015 (0.019) | | -0.016 (0.021) | -0.026 (0.019) |
| *Civic* *engagement* | | |  | |  |  |
| Intercept | 0.098 (0.028) *** | | 0.004 (0.074) | | -0.109 (0.075) | -0.139 (0.066) * |
| Slope | -0.005 (0.007) | | 0.022 (0.014) | | 0.006 (0.016) | -0.003 (0.014) |
| *Social engagement* | | |  | |  |  |
| Intercept | -0.022 (0.025) | | -0.130 (0.055) * | | -0.245 (0.067) *** | -0.096 (0.059) |
| Slope | -0.008 (0.007) | | -0.012 (0.015) | | 0.015 (0.018) | -0.030 (0.015) * |
| *Social activities* |  | |  | |  |  |
| Intercept | -0.050 (0.048) | | -0.234 (0.118) * | | -0.458 (0.121) *** | -0.494 (0.116) *** |
| Slope | -0.023 (0.014) | | -0.015 (0.029) | | 0.002 (0.033) | -0.014 (0.030) |
| *Wish to do more social activities* | |  | |  |  |  |
| Intercept | -0.236 (0.061) *** | | 0.389 (0.150) ** | | 0.119 (0.154) | 0.195 (0.147) |
| Slope | 0.041 (0.018) * | | -0.012 (0.041) | | -0.019 (0.044) | 0.006 (0.041) |

Note: * = *p <* .05, ** = *p* < .01, *** = *p* < .001

Table S8

Regression coefficients for age and trajectory on the intercept and slope for growth models for each index of social and civic engagement for respondents with cancer.

| Effect |  | | Trajectories | | |
| --- | --- | --- | --- | --- | --- |
| Cancer | | Age | | Moderate chronic pain | Fluctuating severe pain |
| *Holidays* | |  | |  |  |
| Intercept | | -0.158 (0.049) *** | | -0.068 (0.143) | -0.229 (0.111) * |
| Slope | | -0.052 (0.014) *** | | -0.037 (0.033) | 0.000 (0.027) |
| *Civic engagement* | |  | |  |  |
| Intercept | | 0.062 (0.046) | | -0.144 (0.133) | -0.219 (0.104) * |
| Slope | | 0.007 (0.012) | | -0.005 (0.028) | -0.014 (0.023) |
| *Social engagement* | |  | |  |  |
| Intercept | | -0.069 (0.039) | | -0.175 (0.110) | -0.166 (0.087) |
| Slope | | -0.012 (0.012) | | 0.003 (0.029) | -0.034 (0.024) |
| *Social activities* | |  | |  |  |
| Intercept | | -0.147 (0.072) * | | -0.021 (0.200) | -0.627 (0.155) *** |
| Slope | | -0.057 (0.019) ** | | -0.027 (0.046) | 0.059 (0.038) |
| *Wish to do more social activities* | |  | |  |  |
| Intercept | | -0.110 (0.087) | | 0.382 (0.241) | 0.407 (0.194) * |
| Slope | | -0.013 (0.025) | | 0.013 (0.063) | -0.003 (0.053) |

Note: * = *p <* .05, ** = *p* < .01, *** = *p* < .001


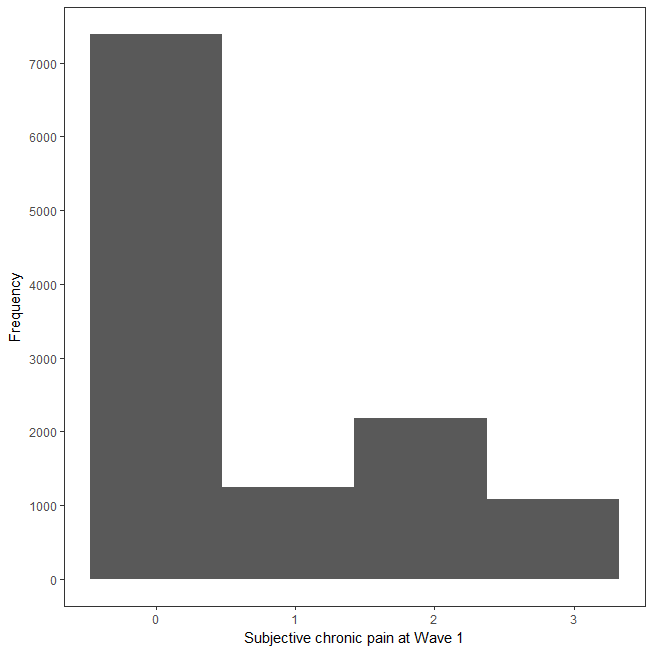


Figure S1

Histogram of the chronic pain measurement at Wave 1 for all Wave 1 ELSA respondents


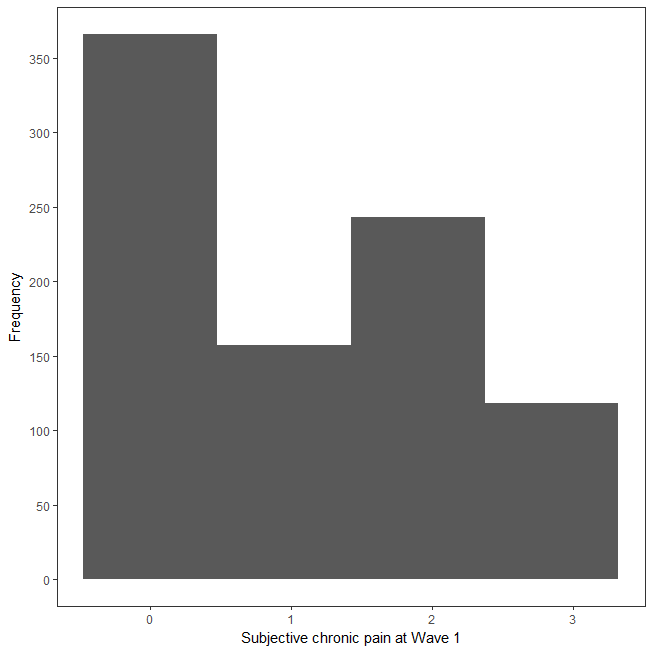


Figure S2

Histogram of the chronic pain measurement at Wave 1 for respondents included in the arthritis subsample.


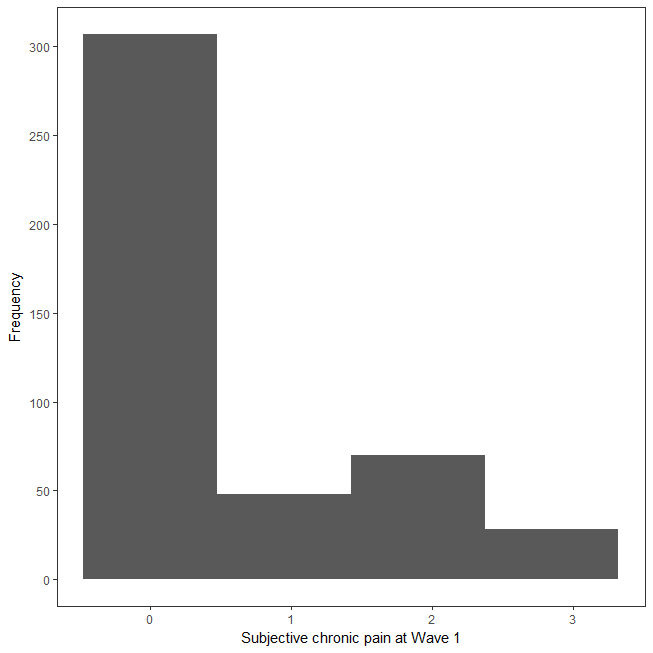


Figure S3

Histogram of the chronic pain measurement at Wave 1 for respondents included in the cancer subsample.
